# *CgNPG1* as a Novel Pathogenic Gene of *Colletotrichum gloeosporioides* From *Hevea brasiliensis* in Mycelial Growth, Conidiation, and the Invasive Structures Development

**DOI:** 10.3389/fmicb.2021.629387

**Published:** 2021-03-08

**Authors:** Chen Liang, Bei Zhang, Yun Zhou, Hongyan Yin, Bang An, Daozhe Lin, Chaozu He, Hongli Luo

**Affiliations:** Hainan Key Laboratory for Sustainable Utilization of Tropical Bioresource, College of Tropical Crops, Hainan University, Haikou, China

**Keywords:** *Colletotrichum gloeosporioides*, *Colletotrichum acutatum*, CgNPG1, mycelial growth, conidiation, pathogenicity

## Abstract

The rubber tree (*Hevea brasiliensis*) is a tropical perennial crop for the primary source of natural rubber. *Colletotrichum gloeosporioides* from *Hevea brasiliensis* (*C. gloeosporioides* Hb) and *Colletotrichum acutatum* from *Hevea brasiliensis* (*C. acutatum* Hb) are the causal agents of rubber tree anthracnose and lead to serious loss of natural rubber production. Inoculation tests showed that *C. gloeosporioides* Hb possessed higher pathogenicity than *C. acutatum* Hb to the rubber tree. Genomic analysis revealed that an unknown gene, named *CgNPG1* (a Novel Pathogenic Gene 1), was presented in the genome of *C. gloeosporioides* Hb but not identified in *C. acutatum* Hb. *CgNPG1* was predicted to encode a small secretory protein without any conserved domain. To investigate the functions of *CgNPG1* in *C. gloeosporioides* Hb and in *C. acutatum* Hb, the gene deletion and overexpression mutants were generated. The phenotype analysis showed that deletion of *CgNPG1* led to changed conidia morphology, decreased mycelial growth, conidiation, conidia germination rate, appressorium formation rate, and pathogenicity of *C. gloeosporioides* Hb to the rubber tree. Meanwhile, heterogeneous expression of *CgNPG1* in *C. acutatum* Hb significantly changed the conidia morphology and improved the mycelial growth rate, conidiation, conidia germination rate, appressorium formation rate, and the pathogenicity of *C. acutatum* Hb to the rubber tree. Consistently, *CgNPG1* increased the expression level of *CaCRZ1* and *CaCMK1* in *C. acutatum* Hb. These data suggested that *CgNPG1* contributed to mycelial growth, conidiation, the development of invasive structures, and the pathogenicity of *Colletotrichum* to the rubber tree, which might be related to the modulation of CaCRZ1 and mitogen-activated protein kinase CMK1. Our results provided new insight into *CgNPG1* in regulating growth and pathogenicity of the *Colletotrichum* spp.

## Introduction

*Colletotrichum* as asexual genus is classified into fungi imperfecti, coelomycetes (Dean et al., 2012). As one of the most common and important genus of filamentous phytopathogenic fungi, *Colletotrichum* caused anthracnose on a wide variety of herbaceous and woody plants in tropical, subtropical, and temperate climates, resulting in serious economic losses (Cannon et al., 2012; Dean et al., 2012; Crouch et al., 2014; Baroncelli et al., 2016). Most *Colletotrichum* species feed on hemibiotrophy, exhibiting initially a brief biotrophic phase with large intracellular primary hyphae and later a destructive, necrotrophic phase with narrower secondary hyphae, which ramify throughout the host tissue (Münch et al., 2008; Dean et al., 2012). Among these *Colletotrichum* species, *Colletotrichum acutatum*, and *Colletotrichum gloeosporioides* are frequently mentioned and reported because of early taxonomic confusion (Dean et al., 2012).

Although multiple inoculation investigations have demonstrated that most isolates of *Colletotrichum* spp. are relatively non-host-specific, there are still some differences in host and tissue pathogenicity between species (Peres et al., 2005; de Souza et al., 2013). For example, *C. acutatum* isolates from pine were non-pathogenic or weakly pathogenic to lupine and vice versa, and the isolates from fruits were not pathogenic on either pine or lupine (Lardner et al., 1999; Peres et al., 2005). In plant pathogens, host specificity is often attributed to pathogen virulence factors, including specialized secondary metabolites (SSM), toxin, cell wall-degrading enzymes, and small-secreted protein (SSP) effectors (Stukenbrock and McDonald, 2007; Brunner et al., 2013; Poppe et al., 2015; Buiate et al., 2017). Recently, several reports have revealed that horizontal gene transfer (HGT) and horizontal chromosome transfer (HCT) between plant pathogenic fungi affect their host range and virulence (Mehrabi et al., 2011; Jaramillo et al., 2014).

Mitogen-activated protein (MAP) kinase signal transduction pathways were involved in fungal pathogenesis (Dickman and Yarden, 1999), and some virulence-involved transcription factors (TFs) played important roles in fungal infection to plant (Dickman and Yarden, 1999; Son et al., 2011). For example, MAP kinase *Cpmk2* from ascomycete *Claviceps purpurea* and *mps1* from *Magnaporthe grisea* contributed to conidiation and virulence (Xu et al., 1998; Mey et al., 2002). MoCRZ1 from rice blast fungus *Magnaporthe oryzae* was essential for cell wall integrity, conidiation, pathogenicity, and Ca^2+^-dependent vegetative growth (Choi et al., 2009). The infection process of *Colletotrichum* includes germination, formation of melanized appressoria, appressorial penetration, and subsequent invasive growth in host plants. In *C. lagenarium*, *CMK1* regulated conidial germination, appressorium formation, and pathogenicity (Takano et al., 2000). In *C. gloeosporioides*, CgCrzA as the homolog of CRZ1, was involved in regulating cell wall integrity and infection-related morphogenesis. Deletion of the *CgCRZA* in *C. gloeosporioides* showed severe cell wall integrity defects and inhibited the vegetative growth, conidiation, appressorial formation rate, and lost pathogenicity to plant hosts (Wang et al., 2020). These data suggested that both CMK1 and CRZ1 in *Colletotrichum* were involved in fungal development and pathogenesis.

The rubber tree (*Hevea brasiliensis*) is a tropical perennial crop for natural rubber; however, rubber latex production is seriously affected by rubber tree anthracnose (Pu et al., 2007; Cao et al., 2017), which are caused by *C. gloeosporioides* and *C. acutatum* (Brown and Soepena, 1994; Saha et al., 2002). In China, *C. gloeosporioides* was considered as the only pathogen of rubber tree anthracnose at first, until *C. acutatum* was first reported as a causal agent of rubber tree anthracnose in Yunnan Province in 2008 and in Hainan in 2010 (Zhang et al., 2008; Li et al., 2010). Further research revealed that both *C. gloeosporioides* and *C. acutatum* can induce typical water-soaked, darker, and circular lesions on rubber tree leaves, but the colony growth rate of *C. gloeosporioides* is significantly higher than that of *C. acutatum* (Cao et al., 2017). So far, little is known about the pathogenic mechanism of *C. gloeosporioides* and *C. acutatum* to rubber trees. In this study, *C. gloeosporioides* Hb and *C. acutatum* Hb were isolated from the leaves of rubber trees infected with anthracnose in Hainan province, and the following analysis showed that *C. gloeosporioides* Hb possesses significantly higher pathogenicity than that of *C. acutatum* Hb to rubber tree leaves. In order to explain the difference of pathogenicity to the rubber tree, the specific genes of *C. gloeosporioides* Hb, but not in *C. acutatum* Hb, were screened, especially *CgNPG1* (a Novel Pathogenic Gene of *C. gloeosporioides*). Further, the biological functions of *CgNPG1* have been characterized through construction of *CgNPG1* deletion mutant in *C. gloeosporioides* Hb and *CgNPG1* heterologous expression in *C. acutatum* Hb. These results not only extend our understanding of the pathogenesis of *C. gloeosporioides* Hb to the rubber tree but also provide novel insight into the host specificity mechanism of *C. gloeosporioides* Hb and *C. acutatum* Hb to the rubber tree.

## Materials and Methods

### Fungal Strains and Culture Conditions

*Colletotrichum gloeosporioides* from *Hevea brasiliensis* and *Colletotrichum acutatum* from *Hevea brasiliensis* strains were isolated from the leaves of *Hevea brasiliensis* with anthracnose in Hainan province. Both strains were grown on potato dextrose agar (PDA) at 28°C in the dark.

### *In silico* Analysis of *CgNPG1*

The amino acid sequence of CgNPG1 was deduced by DNASTAR software. Prediction of signal peptides was performed online by SignalP 5.0 analysis tool^1^. Prediction of conserved domain and motif were performed by Motif Scan^2^. The bootstrap neighbor-joining phylogenetic tree was constructed using Clustal X 2.0 and MEGA X.

### Vector Constructions

For the construction of the gene deletion vector, the 5′ and 3′ flanking region nucleotides of the *CgNPG1* were amplified from genomic DNA and ligated into the vector pCB1532 carrying the acetolactate synthase gene (SUR) cassette from *M. oryzae* conferred resistance to chlorimuron ethyl (a sulfonylurea herbicide) (Supplementary Figure 1A). For construction of the complementation vector, the open read frame of *CgNPG1* was amplified from cDNA, fused with the 3 X FLAG coding sequence, and cloned into the vector harboring the promoter of *ToxA*, the terminator of nos, and the hygromycin phosphotransferase gene (HPH) (Supplementary Figure 2A).

### Transformation of *Colletotrichum gloeosporioides* and *Colletotrichum acutatum*

Protoplast preparation and transformation were carried out as described in our previous work (Wang et al., 2018). The deletion mutants were analyzed by two round PCR analyses, which were diagnostic tests for homologous integration of the 5′ part and 3′ part. Then the correct transformants were purified by single conidia isolations and analyzed by Southern blot. For Southern blot analysis, genomic DNA of wild type and the deletion mutant was extracted and digested with *HindIII*, and the upstream flanking region of *CgNPG1* was amplified and used as probe. Another PCR was conducted for screening existence of *CgNPG1*. The complementation mutant of *C. gloeosporioides* Hb and the heterogeneous expression mutant of *C. acutatum* Hb were identified by PCR analysis, and the *CgNPG1* expression level of the mutants was estimated by RT-PCR.

### RNA Isolation, cDNA Synthesis, and qRT-PCR

RNA was extracted from the mycelium of *C. gloeosporioides* Hb and *C. acutatum* Hb using the CTAB-LiCl method (Yang et al., 2020). The contaminating DNA was eliminated using RNase-free DNase, and the first-strand cDNA was synthesized using Revert Aid First Strand cDNA Synthesis Kit (Thermo Fisher). Quantitative RT-PCR analysis was performed with the LightCycler 96 System (Roche). The beta-tubulin-1 (β-tub1) gene was used as an endogenous control for normalization. Relative expression levels of target genes were estimated using the 2^–ΔΔ^ ^Ct^ method.

### Fungal Growth Assay

For the vegetate growth assay, 5-mm-diameter disks of mycelium together with agar were inoculated on fresh potato dextrose agar medium and cultured for 9 days. The diameters of the colonies were recorded, and the growth rates were calculated. The experiment was repeated three times. Statistical analysis was performed by SPSS software (version 20), with *P* < 0.05 as statistically significant.

### Conidiation and Appressorium Formation Assay

For the conidiation assay, conidia were harvested from the strains growing on PDA medium for 8 days and inoculated into 50-ml liquid CMC medium to the final concentration of 10^4^/ml, respectively. Then all samples were cultured at 28°C, 150 rpm, and the conidia numbers were calculated under a microscope after incubation for 2 days.

For appressorium formation assays, 20-μl drops of conidial suspensions were placed on nylon membrane and incubated at 28°C. After 4 and 8 h post-incubation, the percentages of conidial germination and appressorium formation were determined under a microscope, respectively. The experiment was repeated three times, and at least 100 conidia were detected per replicate.

### Pathogenicity Test

For the pathogenicity test, conidia suspension was used to inoculate the rubber tree variety 7–33–97 leaves at the “light green” stage. Conidia were harvested from mycelium grown on potato dextrose agar medium for 12 days in a 28°C incubator, washed with double-distilled H_2_O, filtered through a filter membrane (Miracloth, Millipore), and resuspended in a solution of 5% Sabouraud Maltose Broth (Difco) to a final concentration of 2 × 10^5^ conidia/ml. Then 10 μl of the conidial suspensions were inoculated onto the wounded rubber tree leaves. The inoculated leaves were kept in a moist chamber at 28°C under natural illumination for 4 days, and the disease symptoms were scored. Each treatment contained three replicates of nine leaves, and the entire experiment was repeated three times. Statistical analysis was performed by SPSS software (version 20), with *P* < 0.05 as statistically significant.

## Results

### Pathogenicity Difference of *Colletotrichum gloeosporioides* Hb and *Colletotrichum acutatum* Hb on Rubber Tree Leaves

**FIGURE 1:**
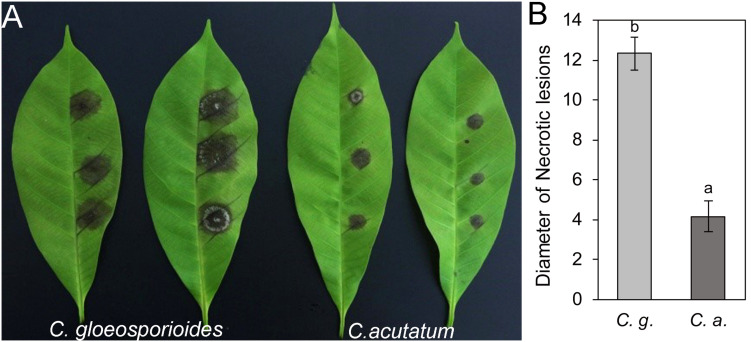
Pathogenicity assay of *Colletotrichum gloeosporioides* Hb and *Colletotrichum acutatum* Hb on rubber tree leaves. **(A)** Disease symptoms of rubber-tree leaves at 3 days post-inoculation with *C. gloeosporioides* Hb and *C. acutatum* Hb mycelium. **(B)** Lesion diameters at 3 day post-inoculation. Error bars indicated standard deviation, and columns with different letters indicate significant difference (*P* < 0.05).

*Colletotrichum gloeosporioides* from *Hevea brasiliensis* and *Colletotrichum acutatum* from *Hevea brasiliensis* were isolated and characterized from rubber trees in Hainan province of China. When the detached “light green” rubber tree leaves from variety 73–3–97 were inoculated with *C. gloeosporioides* Hb and *C. acutatum* Hb, respectively, the necrotic lesions were obviously observed on the 4th day (Figure 1A). The mean size of the disease lesion was about 1.3 cm in *C. gloeosporioides* Hb, while that of *C. acutatum* Hb was only about 0.4 cm (Figure 1B), suggesting that *C. gloeosporioides* Hb possesses higher pathogenicity to the rubber tree than *C. acutatum* Hb.

### Cloning and Analysis of Specific Pathogenic Genes of *Colletotrichum gloeosporioides* Hb

**FIGURE 2:**
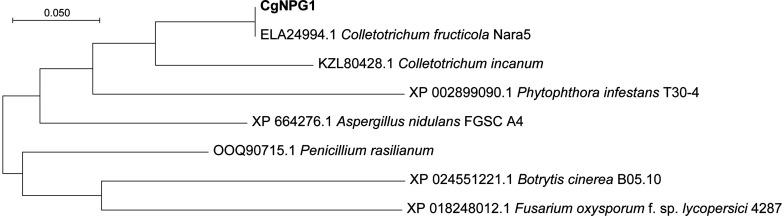
Phylogenetic analysis of a Novel Pathogenic Gene 1 (CgNPG1). Phylogenetic analysis of CgNPG1 and another seven orthologs by neighbor-joining methods using MEGA X software.

Genome sequencing of *C. gloeosporioides* Hb and *C. acutatum* Hb was produced by Illumina Hiseq2000. Both of the genome sequences have been uploaded to the NCBI database (Bioproject ID: PRJAN690880). In order to reveal the mechanism of the different pathogenicities of *C. gloeosporioides* Hb and *C. acutatum* Hb to the rubber tree, the genes encoding secretory proteins were predicted. Among these genes, three genes encoded proteins with no putative conserved domains and were specific to *C. gloeosporioides* Hb than to *C. acutatum* Hb. One of them was named *CgNPG1* (a Novel Pathogenic Gene of *C. gloeosporioides* Hb). *CgNPG1* contains a 435-bp open reading frame encoding a polypeptide of 144 amino acids with a molecular weight of approximately 15.0 kDa and a theoretical pI of 4.28. The CgNPG1 protein contained 10 cysteines, accounting for 6.9% of the total number of amino acids. Signal peptide was predicted at the N-terminal of 1 to 16 amino acids (Supplementary Figure 1). In addition, the transmembrane domain and any known conserved domains were not found in the CgNPG1 protein. In addition, the protein sequences of CgNPG1 and some highly homologous protein sequences of representative hemibiotrophic fungi, necrotrophic fungi, and oomycetes from Blastp were used to generate a phylogenetic tree. Phylogenetic tree analysis showed that the homologous proteins of CgNPG1 widely exist in hemibiotrophic fungi, necrotrophic fungi, and oomycetes, and CgNPG1 is the closest, homologous to *Colletotrichum* and oomycetes (Figure 2).

### Generation of *CgNPG1* Deletion and Complementary Mutant in *Colletotrichum gloeosporioides*, and Heterogeneous Expression Mutant in *Colletotrichum acutatum*

To investigate the functions of *CgNPG1* in *C. gloeosporioides* Hb, the *CgNPG1* gene deletion and complementation mutants were generated. In addition, the *CgNPG1* heterogeneous expression mutant of *C. acutatum* Hb was generated to investigate its roles on *C. acutatum* Hb. The *CgNPG1* deletion mutant of *C. gloeosporioides* Hb (Cg-Δ*NPG1*) was generated by homologous recombination strategy (Supplementary Figure 2A). PCR diagnostic analysis showed that *CgNPG1* was successfully deleted from the *C. gloeosporioides* Hb genome (Supplementary Figure 2B). Southern blot assays revealed that the *CgNPG1* had only one copy and was deleted in the Cg-Δ*NPG1* mutant (Supplementary Figure 2C). The complementation strain (Cg-ResΔ*NPG1*) was generated by expressing *CgNPG1* driven by a ToxA promoter in the Cg-Δ*NPG1* mutant (Supplementary Figure 3A). PCR and semi-quantitative RT-PCR analysis showed that *CgNPG1* was restored in the complementary strain (Supplementary Figures 3B,C). The complementary vector of *CgNPG1* was used to transform *C. acutatum* Hb to generate the heterogeneous expression mutant (Ca-*NPG1*). PCR and semiquantitative RT-PCR analysis showed that *CgNPG1* was successfully integrated into the genome and expressed in *C. acutatum* Hb (Supplementary Figure 4).

### *CgNPG1* Contributes to Colony Growth of *Colletotrichum gloeosporioides* Hb and *Colletotrichum acutatum* Hb

**FIGURE 3:**
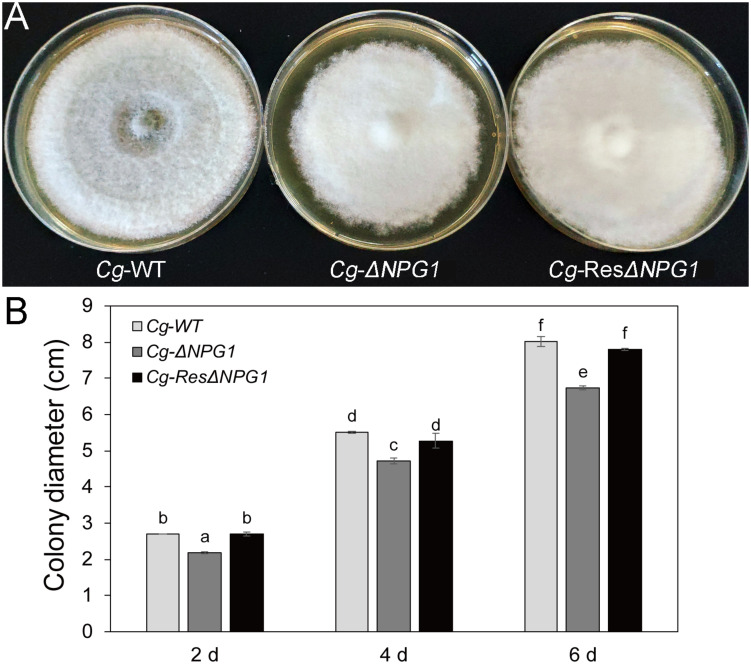
Growth rate assays of *CgNPG1* deletion and complementation mutants of *C. gloeosporioides* Hb. **(A)** Colonial morphology of wild type strain (Cg-WT), the *CgNPG1* deletion mutant (Cg-ΔNPG1), and complementation mutant (Cg-ResΔNPG1) after culture for 8 day on potato dextrose agar. **(B)** Colony diameters at 2, 4, and 6 day post-inoculation. Error bars indicated standard deviation, and columns with different letters indicate significant difference (*P* < 0.05).

**FIGURE 4:**
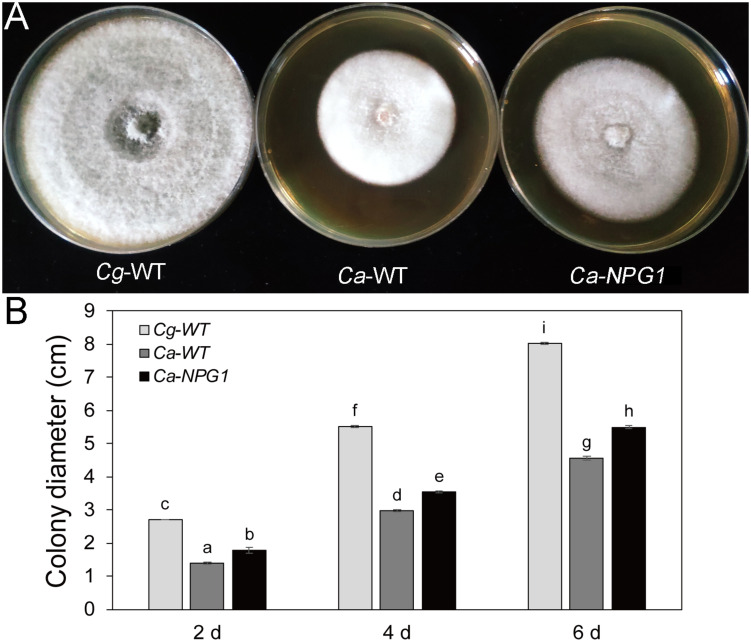
Growth rate assays of *C. acutatum* Hb strains expressing *CgNPG1*. **(A)** Colonial morphology of *C. gloeosporioides* Hb wild type strain (Cg-WT), *C. acutatum* Hb wild type strain (Ca-WT), and the *CgNPG1* heterologous expression mutant (Ca-*NPG1*). **(B)** Colony diameters at 2, 4, and 6 day post-inoculation. Error bars indicated standard deviation, and columns with different letters indicate significant difference (*P* < 0.05).

Growth analysis showed that the deletion of *CgNPG1* obviously decreased the colony growth rate in *C. gloeosporioides* Hb, with the colony diameter decreased to 6.5 cm in Cg-Δ*NPG1* in comparison to 8 cm in Cg-WT at 8 days after inoculation. Meanwhile, the Cg-Δ*NPG1* showed different colonial morphology with more aerial hyphae compared with Cg-WT. In addition, complementation of *CgNPG1* restored the growth rate of Cg-Δ*NPG1* (Figure 3). Heterologous expression of *CgNPG1* significantly promoted the colony growth rate in *C. acutatum* Hb (Figure 4). These results suggested that CgNPG1 contributed to the vegetative growth of *C. gloeosporioides* Hb and *C. acutatum* Hb.

### *CgNPG1* Contributes to Conidia Morphology and Conidiation of *C. gloeosporioides* Hb and *C. acutatum* Hb

**FIGURE 5:**
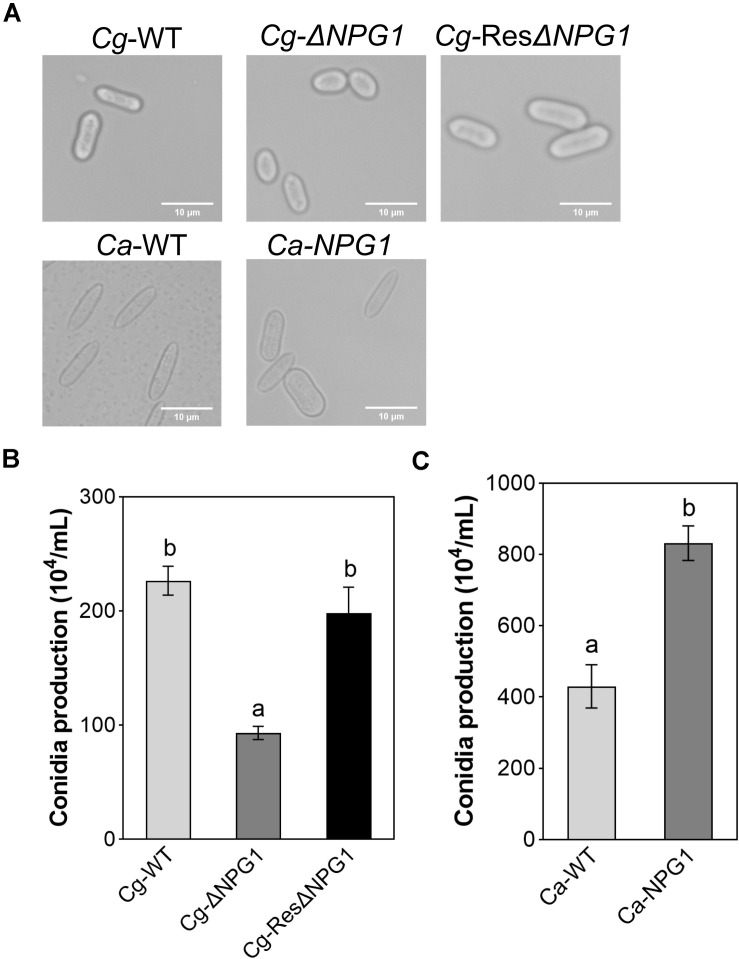
Conidia morphology observation and conidiation assay of *CgNPG1* deletion mutant, *CgNPG1* complementation mutant, and *CgNPG1* heterologous expression mutant. **(A)** Conidial morphology of Cg-WT, Cg-Δ*NPG*, Cg-ResΔ*NPG1*, Ca-WT, and Ca-*NPG1.* **(B)** The number of conidia developed by the Cg-WT, Cg-Δ*NPG*, Cg-ResΔ*NPG1*. **(C)** The number of conidia developed by the Ca-WT and Ca-*NPG1*. Error bars indicated standard deviation, and columns with different letters indicate significant difference (*P* < 0.05).

The morphological observation showed that the Conidia of Cg-Δ*NPG1* were significantly shorter than that of Cg-WT and complemented strains Cg-ResΔ*NPG1*, and heterologous expression of *CgNPG1* in *C. acutatum* Hb caused more than 65% of conidia to become blunt round at both ends and fatter compared with Ca-WT (Figure 5A). Conidiation assay showed that the conidia production of Δ*CgNPG1* was drastically reduced to less than half of Cg-WT and Cg-ResΔ*NPG1* (Figure 5B), and heterologous expression of *CgNPG1* in *C. acutatum* Hb doubled the conidia yield of Ca-WT (Figure 5C).

### *CgNPG1* Is Involved in Conidia Germination and Appressorium Formation

**FIGURE 6:**
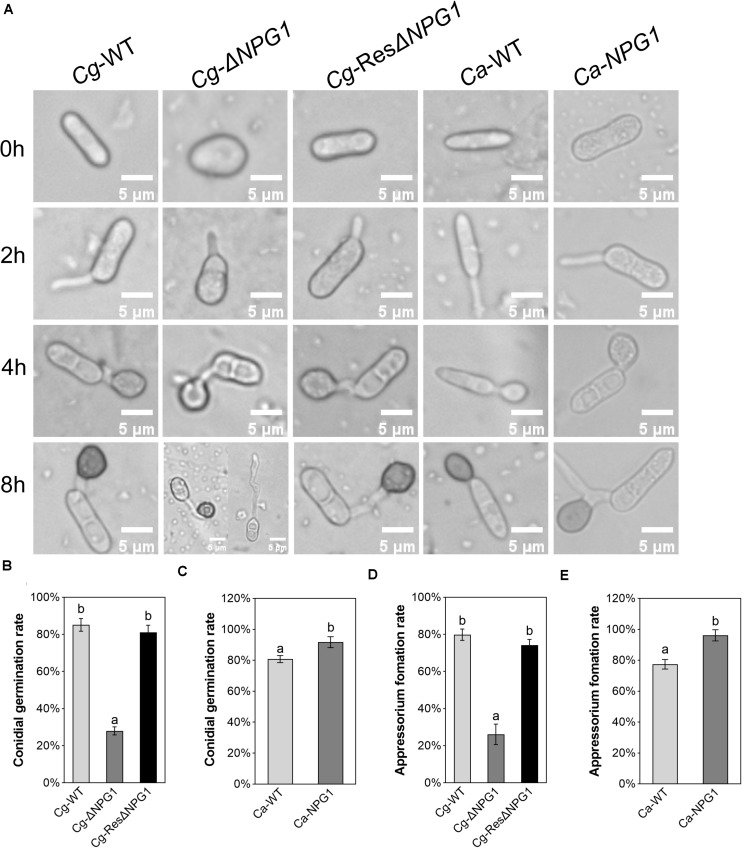
Conidial germination and appressorium formation assay of *CgNPG1* deletion mutant and *CgNPG1* heterologous expression mutant. **(A)** Appressorium development progress of Cg-WT, Cg-Δ*NPG*, Cg-ResΔ*NPG1*, Ca-WT, and Ca-*NPG1*. **(B)** Conidia germination rates of Cg-WT, Cg-Δ*NPG*, Cg-ResΔ*NPG1* at 4 h time intervals. **(C)** Conidia germination rates of Ca-WT and Ca-*NPG1* at 4 h time intervals. **(D)** Appressorium formation rates of Cg-WT, Cg-Δ*NPG*, Cg-ResΔ*NPG1* at 8 h time intervals. **(E)** Appressorium formation rates of Ca-WT and Ca-*NPG1* at 8 h time intervals. Error bars indicated standard deviation, and columns with different letters indicate significant difference (*P* < 0.05).

The processes of conidia germination and appressorium formation was observed and counted on nylon membrane. In Cg-WT and Cg-ResΔ*NPG1*, more than 80% of the conidia germinated after 4 h of post-incubation, compared with less than 30% in Cg-Δ*NPG1* (Figures 6A,B). In the Cg-Δ*NPG1*mutant, over 70% of conidia failed to form abnormal appressoria after 8 h of post-incubation, compared with Cg-WT and Cg-ResΔ*NPG1* strains, which formed normal appressoria (Figures 6A,D). Additionally, after 4 h of post-incubation, the conidia germination rate of Ca-*NPG1* strain was about 90%, which was significantly higher than that of wild type Ca-WT that was about 80% (Figure 6C). After 8 h of post-incubation, the appressorium information rate of Ca-*NPG1* strain was more than 95%, which was significantly higher than that of the wild type Ca-WT strain, which was nearly 80% (Figure 6E). These data indicated that *CgNPG1* was involved in the invasive structure development of *C. gloeosporioides* Hb, including conidia germination and normal appressorium formation, and heterologous expression of *CgNPG1* in *C. acutatum* Hb promoted conidia germination and appressorium formation.

### *CgNPG1* Is Required for the Virulence of *Colletotrichum gloeosporioides* Hb and Increases the Virulence of *Colletotrichum acutatum* Hb on Rubber Tree

**FIGURE 7:**
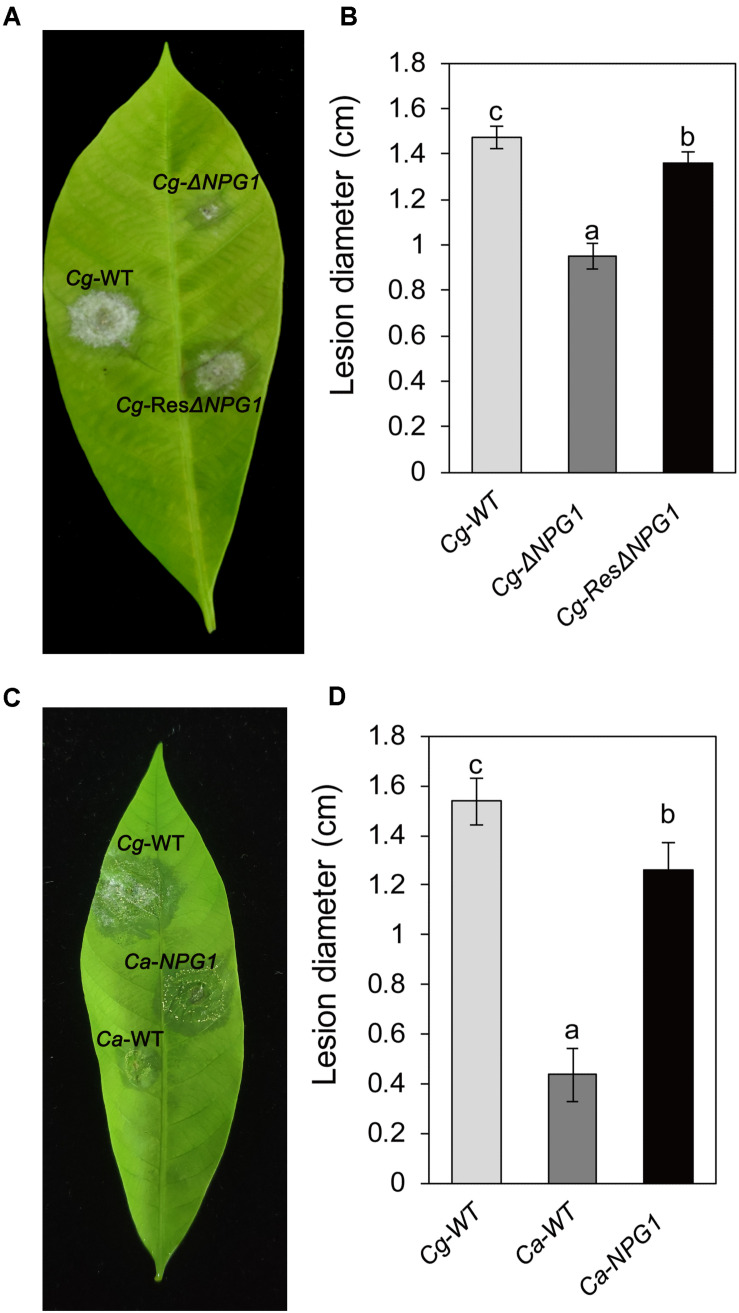
Pathogenicity assay of *CgNPG1* deletion mutant and *CgNPG1* heterologous expression mutant on rubber tree leaves. **(A)** Disease symptoms of rubber tree leaves at 4 days post-inoculation with conidia of *C. gloeosporioides* Hb wild type (Cg-WT), the *CgNPG1* deletion mutant (Cg-Δ*NPG1*), and complementation mutant (Cg-ResΔ*NPG1*) strains. **(B)** Lesion diameters at 4 day post-inoculation with conidia of Cg-WT, Cg-Δ*NPG1*, and Cg-ResΔ*NPG1* strains. **(C)** Disease symptoms of rubber tree leaves at 4 days post-inoculation with conidia of *C. gloeosporioides* Hb wild type (Cg-WT), *C. acutatum* Hb wild type (Ca-WT), and the *CgNPG1* heterologous expression mutant (Ca-*NPG1*) strains. **(D)** Lesion diameters at 4 day post-inoculation with conidia of Cg-WT, Ca-WT, and Ca-*NPG1* strains. Error bars indicated standard deviation, and columns with different letters indicate significant difference (*P* < 0.05).

Detached rubber tree leaves were used for pathogenicity analysis. After inoculation for 4 day, both Cg-Δ*NPG1* and Cg-ResΔ*NPG1* caused typical anthracnose symptoms as well as Cg-WT (Figure 7A), but the mean lesion diameter caused by Cg-Δ*NPG1* (only 0.9 cm) was obviously smaller than that caused by Cg-WT (over 1.4 cm) (Figure 7B). Meanwhile, Cg-ResΔ*NPG1* basically restored the pathogenicity of Cg-Δ*NPG1*, with a lesion diameter of 1.3 cm after inoculation at 4 days. Interestingly, heterologous expression of *CgNPG1* in *C. acutatum* Hb significantly increase the pathogenicity of *C. acutatum* Hb to rubber tree leaves, with lesion diameters of 1.3 cm in Ca-*NPG1*; in contrast, the mean lesion diameter of Ca-WT was only 0.4 cm (Figures 7C,D). These data demonstrated that *CgNPG1* play important roles in the pathogenicity of *C. gloeosporioides* Hb and *C. acutatum* Hb to rubber tree.

### *CgNPG1* Increased the Expression of *CaCRZ1* and *CaCMK1* in *Colletotrichum acutatum* Hb

**FIGURE 8:**
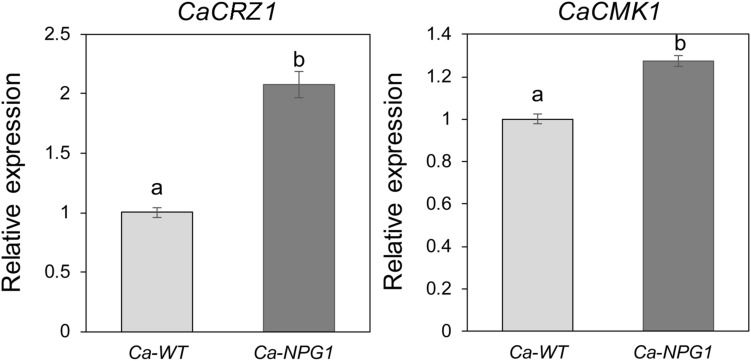
Quantitative RT-PCR analysis. Relative expression levels of *CaCRZ1* and *CaCMK1* were estimated using the 2^− ΔΔ^ ^Ct^ method with *beta-tubulin* 1 as the reference gene. Error bars indicated standard deviation, and columns with different letters indicate significant difference (*P* < 0.05).

CRZ1 and CMK1 act as regulators of the upstream signals to control the growth, conidiation, invasive structures development, and pathogenicity in fungal pathogens (Mey et al., 2002; Wang et al., 2020). Here, heterologous expression of *CgNPG1* in *C. acutatum* Hb obviously upregulated the transcription of *CaCRZ1* and *CaCMK1*, with the expression level of *CaCRZ1* increased to twofold and *CaCMK1* to 1.3-fold (Figure 8), indicating that the effect of *CgNPG1* on *C. acutatum* Hb might be related to the modulation of CaCRZ1 and CaCMK1.

## Discussion

*Colletotrichum* spp. is one of the most common and important genera of plant pathogenic fungi, causing anthracnose on important staple food crops (Dean et al., 2012). Although both *C. gloeosporioides* and *C. acutatum* could cause typical anthracnose on rubber tree (*Hevea brasiliensis*) (Zhang et al., 2008; Li et al., 2010), there is virulence difference between the two pathogens. Consistently, the anthracnose caused by *C. gloeosporioides* Hb was more severe than that by *C. acutatum* Hb, suggesting that *C. gloeosporioides* Hb possesses higher pathogenicity. For the same race of plant pathogens, individual pathogenic isolates possesses different abilities to infect just one or a few host species, and these different isolates are conventionally merged into infraspecific assemblages, known as formae speciales (ff. spp.). Different isolates of the same formae speciales could be further separated into different virulence groups. Genomic analysis revealed that the weakly virulent isolates often lack some virulence factors, including TFs, effectors, cell wall-degrading enzymes, and toxins (Stukenbrock and McDonald, 2007; de Vega-Bartol et al., 2011; Brunner et al., 2013; Poppe et al., 2015; Buiate et al., 2017). Notably, the presence of particular small secreted proteins is associated with the determination of host range in some phytopathogenic fungi, including *Alternaria* spp., *Cochliobolus* spp., and *Fusarium* spp. (Ito et al., 2004; Houterman et al., 2008; Bhadauria et al., 2015). Therefore, the objective of this study was to investigate whether there are special virulence factors that contribute to the virulence difference between *C. gloeosporioides* Hb and *C. acutatum* Hb, especially *CgNPG1* as a potential specific pathogenic factor in *C. gloeosporioides* Hb. Although no conserved domains was identified in CgNPG1, phylogenetic tree analysis showed that the homologous proteins of CgNPG1 widely existed in plant pathogens, including the most common fungal pathogens and oomycetes (*Phytophthora infestans*), and human pathogens (*Aspergillus nidulans* and *Penicillium brasilianum*) (Figure 2). CgNPG1 contained 144 amino acids, of which 10 cysteines accounted for 6.9% and did not have any transmembrane domain except a signal peptide at the N-terminal of 1 to 16 amino acids (Supplementary Figure 1). Usually, the SSPs classified as effectors had the following characteristics: the sequence length of ≤300 amino acids and ≥4 cysteines, and function as key infection factors in suppressing host defense responses and modulating its physiology (Feldman et al., 2020). In our study, CgNPG1 matched the above sequence characteristics and effects in mycelial growth, conidiation, invasive structure development, and pathogenesis (Figures 3–7). Moreover, the prediction results by EeffectorP1.0 software showed that CgNPG1 was an effector. Based on the above data, CgNPG1 could be considered as an effector.

To investigate the function of *CgNPG1*, its nucleotide sequence was deleted from the genome of *C. gloeosporioides* Hb and heterogeneously expressed in *C. acutatum* Hb. *In vitro* test showed that mycelial growth of Cg-Δ*NPG1* mutant on PDA medium decreased slightly but was still comparable to that of the wild type, and growth rate recovered to the wild type level in the restored transformants (Figure 3). Heterogeneous expression of *CgNPG1* in *C. acutatum* Hb obviously increased the colony growth rate (Figure 4). Leaf inoculation tests showed that deletion of *CgNPG1* in *C. gloeosporioides* Hb resulted in significant decreased pathogenicity. Although Cg-Δ*NPG1* mutant could infect the leaves and develop necrotic lesions, but the lesion diameters decreased by about 50% compared to that of the WT and the restored transformants (Figures 7A,B). Heterogeneous expression of *CgNPG1* in *C. acutatum* Hb significantly increased the pathogenicity of *C. acutatum* Hb to the rubber tree (Figures 7C,D), with the disease severity of *Ca-NPG1* increased by about 60% compared with the Ca-WT strain. Interestingly, the deletion of *CgNPG1* in *C. gloeosporioides* Hb decreased the conidiation and changed the conidia morphology, and heterogeneous expression of *CgNPG1* in *C. acutatum* Hb increased the conidiation and also changed the morphology. In addition, we also found that the deletion of *CgNPG1* could affect conidia germination and appressorium formation (Figure 6). Since *CgNPG1* was required for the mycelial growth, conidiation, conidia morphology, and germination, and pathogenicity of *C. gloeosporioides* Hb and *C. acutatum* Hb, the deletion of *CgNPG1* in *C. acutatum* Hb might contribute to the poor pathogenicity to the rubber tree, at least partially.

Specialization to a new host is possible through advantageous mutations, vertical inheritance, and gene transfer events. Gene transfer (HGT and HCT) is associated with host specificity (Jaramillo et al., 2014). In *Fusarium oxysporum*, transfer of lineage-specific chromosomes between strains by HCT could even convert a non-pathogenic strain into a pathogen (Ma et al., 2010). For HGT, the candidate transferred genes are those constantly subject to gene duplication and gene loss. In *Pezizomycotina*, although no evidence of a burst of HGT events coinciding with major geological events was found, HGT appears to be occurring at a steady rate during their evolution (Jaramillo et al., 2014). Between two vegetative incompatible biotypes of *C. gloeosporioides*, it has been proved that HCT can occur during co-cultivation under laboratory conditions and most likely in nature (Masel et al., 1996; He et al., 1998). Moreover, 11 HGT events from bacteria into *Colletotrichum* or their ancestors have been identified, and some of the genes play roles in virulence (Jaramillo et al., 2014). In our case, to explore the possibility of HGT event in *Colletotrichum*, we constructed the phylogenetic relationships among the fungi close to *C. gloeosporioides* based on their 28S ribosomal sequences (Supplementary Figure 5). Referring to the phylogenetic tree, none homolog of *NPG1* was further found in these close organisms other than *Colletotrichum.* However, the high similarity was found between *NPG1* genes in *Colletotrichum* and *Phytophthora* (Supplementary Figure 6). Collectively, the results indicated the occurrence of HGT events.

Perception of environmental signals initiates intracellular signal transduction pathways and promote infection-related morphogenesis in phytopathogens. Ca^2+^-calmodulin signal transduction pathways controls a wide array of processes in cell growth and development (Berridge et al., 2003) through the mediation of transcription factors (Cyert, 2003). As a major component of Ca^2+^ signaling pathway, MoCRZ1 was involved in growth, conidiation, and pathogenicity (Choi et al., 2009). As CRZ1 homolog in *C. gloeosporioides* s.s. strain SMCG1#C, CgCrzA was also involved in vegetative growth, conidiation, appressorial formation rate, and lost pathogenicity to plant hosts (Wang et al., 2020). In addition, MAP kinase cascades are also an important regulatory system in cells (Dean, 1997). In *C. lagenarium*, CMK1 regulates the conidiation, appressoria formation, and pathogenic growth (Takano et al., 2000). Here, our results showed that the mutant heterogeneously expressing *CgNPG1* (Ca-*NPG1*) exhibited changed conidia morphology (Figure 5A), enhanced mycelia growth rate (Figure 4), conidiation (Figure 5C), conidia germination rate, appressorial formation rate (Figures 6C,E), and pathogenicity to rubber trees (Figures 7C,D). These results suggested that the function of CgNPG1 was similar to the function of CgCrzA and CMK1. Besides that, our results also showed that heterogeneous expression of *CgNPG1* significantly increased the expression level of *CaCRZ1* and induced the transcription of *CaCMK1* in *C. acutatum* Hb (Figure 8), suggesting that the regulation of *CgNPG1* on *C. acutatum* Hb might be related to *CaCRZ1* and *CaCMK1*.

Taken together, these data suggested that *C. gloeosporioides* Hb-specific *CgNPG1* played important roles in controlling mycelial growth, conidiation, and the development of invasive structures, such as conidia morphology and germination, appressorium formation, and pathogenicity.

## Data Availability Statement

The original contributions presented in the study are included in the article/Supplementary Material, further inquiries can be directed to the corresponding author.

## Author Contributions

CL carried out most of the experiments and analyzed the data. BZ carried out the complementation experiments and the data assay. YZ cloned the *CgNPG1* gene. HY completed the bioinformatics analysis of HGT. CL, BA, and HL wrote the manuscript. CH revised the manuscript. All authors read and approved the final manuscript.

## Conflict of Interest

The authors declare that the research was conducted in the absence of any commercial or financial relationships that could be construed as a potential conflict of interest.

## Footnotes

1 http://www.cbs.dtu.dk/services/SignalP/

2 http://myhits.isb-sib.ch/cgi-bin/motif_scan

## Supplementary Material

Supplementary Figure 1 Nucleotide sequence and deduced amino acid sequence of *CgNPG1*. The predicted signal peptide is shaded by medium gray.

Supplementary Figure 2 Generation and molecular confirmation of *CgNPG1* deletion mutant (Cg-Δ*NPG1*). **(A)** The gene deletion strategy. **(B)** Diagnostic PCR analysis for correct integration of recombinant fragment into the *CgNPG1* locus. **(C)** Southern blot analysis of wild type and Cg-Δ*NPG1* mutant.

Supplementary Figure 3 Generation and molecular confirmation of *CgNPG1* complementation mutant (Cg-*Res*Δ*NPG1*). **(A)** The diagram of complementation vector. **(B)** Diagnostic PCR analysis for integration of *CgNPG1* into the genome of the *CgNPG1* deletion mutant. **(C)** Semi-quantitative RT-PCR analysis of *CgNPG1* expression level in Cg-ΔNPG1 and Cg-ResΔ*NPG1*.

Supplementary Figure 4 Generation and molecular identification of mutant with heterogenous Expression of *CgNPG1* in *C. acutatum* Hb (Ca-*NPG1*). **(A)** Diagnostic PCR analysis for integration of *CgNPG1* open read frame into the genome of *C. acutatum* Hb. **(B)** Semi-quantitative RT-PCR analysis of *CgNPG1* expression level in Ca-*NPG1*strains.

Supplementary Figure 5 Phylogenetic tree of top 600 fungi with close relationships to *C. gloeosporioides* Hb utilizing 28 s sequences (Ribosomal Database Project, Release 11, http://rdp.cme.msu.edu).

Supplementary Figure 6 Phylogenetic tree of top 10 sequences with close relationships to *CgNPG1*.
