# Supplementary material for: CgNPG1 as a Novel Pathogenic Gene of Colletotrichum gloeosporioides From Hevea brasiliensis in Mycelial Growth, Conidiation, and the Invasive Structures Development
Source: Front Microbiol. 2021 Mar 8;12:629387. doi: 10.3389/fmicb.2021.629387 (PMC7982478; doi:10.3389/fmicb.2021.629387)
Supplement: Supplementary Figure 1 — Nucleotide sequence and deduced amino acid sequence of CgNPG1. The predicted signal peptide is shaded by medium gray. [file Data_Sheet_1.PDF]

```
         10        20        30        40        50        60        70        80        90
1   ATGCAGTTCGCCTACTTCCTCCTCGCCGCCCCCGCCCTCGTGGCCGCGACCCTCGACCCGGCCACCTCCAACACCAAGGGCGCCTGCCCG
1    M  Q  F  A  Y  F  L  L  A  A  P  A  L  V  A  A  T  L  D  P  A  T  S  N  T  K  G  A  C  P

        100       110       120       130       140       150       160       170       180
91  AGCGTCTACAACTGCAGCGCGACCAAGGTGTCCAAGGCCATCCAGGCCGCCGAGTGCTCGCACAACACGCGCACCTCCAAGACGCAGACC
31   S  V  Y  N  C  S  A  T  K  V  S  K  A  I  Q  A  A  E  C  S  H  N  T  R  T  S  K  T  Q  T

        190       200       210       220       230       240       250       260       270
181 TTTGCCGTCTTCGAGACGGACCACCAGTACGACGGCAACAACGGCGCCCCCTACGGCACCTGCTCCGCGTACACCTGCGATCCGCCCACG
61   F  A  V  F  E  T  D  H  Q  Y  D  G  N  N  G  A  P  Y  G  T  C  S  A  Y  T  C  D  P  P  T

        280       290       300       310       320       330       340       350       360
271 AGCTCGCAGATGACGACTGACGCGGACTGCTGGACCTTTTTCTGGAGTGGCGAGGGAACTTCTTCTGGCGAGGGCGCTGGATGCATCAAG
91   S  S  Q  M  T  T  D  A  D  C  W  T  F  F  W  S  G  E  G  T  S  S  G  E  G  A  G  C  I  K

        370       380       390       400       410       420       430
361 GACCCCAACACCGGCGAGTGTGGCTGCGAGAACTCTGATGGAACCTTCGTTCCTGGCAGCGATAGCTGTGTTTAA
121  D  P  N  T  G  E  C  G  C  E  N  S  D  G  T  F  V  P  G  S  D  S  C  V  *
```
