# Supplementary figures and images for: CgNPG1 as a Novel Pathogenic Gene of Colletotrichum gloeosporioides From Hevea brasiliensis in Mycelial Growth, Conidiation, and the Invasive Structures Development
Source: Front Microbiol. 2021 Mar 8;12:629387. doi: 10.3389/fmicb.2021.629387 (PMC7982478; doi:10.3389/fmicb.2021.629387)

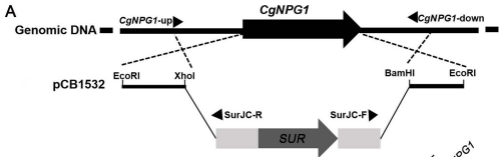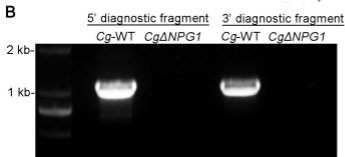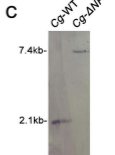

Supplement: Supplementary Figure 2 — Generation and molecular confirmation of CgNPG1 deletion mutant (Cg-ΔNPG1). (A) The gene deletion strategy. (B) Diagnostic PCR analysis for correct integration of recombinant fragment into the CgNPG1 locus. (C) Southern blot analysis of wild type and Cg-ΔNPG1 mutant. [file Data_Sheet_2.PDF]

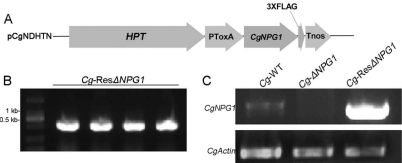

Supplement: Supplementary Figure 3 — Generation and molecular confirmation of CgNPG1 complementation mutant (Cg-ResΔNPG1). (A) The diagram of complementation vector. (B) Diagnostic PCR analysis for integration of CgNPG1 into the genome of the CgNPG1 deletion mutant. (C) Semi-quantitative RT-PCR analysis of CgNPG1 expression level in Cg-ΔNPG1 and Cg-ResΔNPG1. [file Data_Sheet_3.PDF]

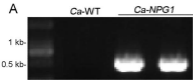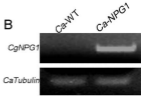

Supplement: Supplementary Figure 4 — Generation and molecular identification of mutant with heterogenous Expression of CgNPG1 in C. acutatum Hb (Ca-NPG1). (A) Diagnostic PCR analysis for integration of CgNPG1 open read frame into the genome of C. acutatum Hb. (B) Semi-quantitative RT-PCR analysis of CgNPG1 expression level in Ca-NPG1strains. [file Data_Sheet_4.PDF]

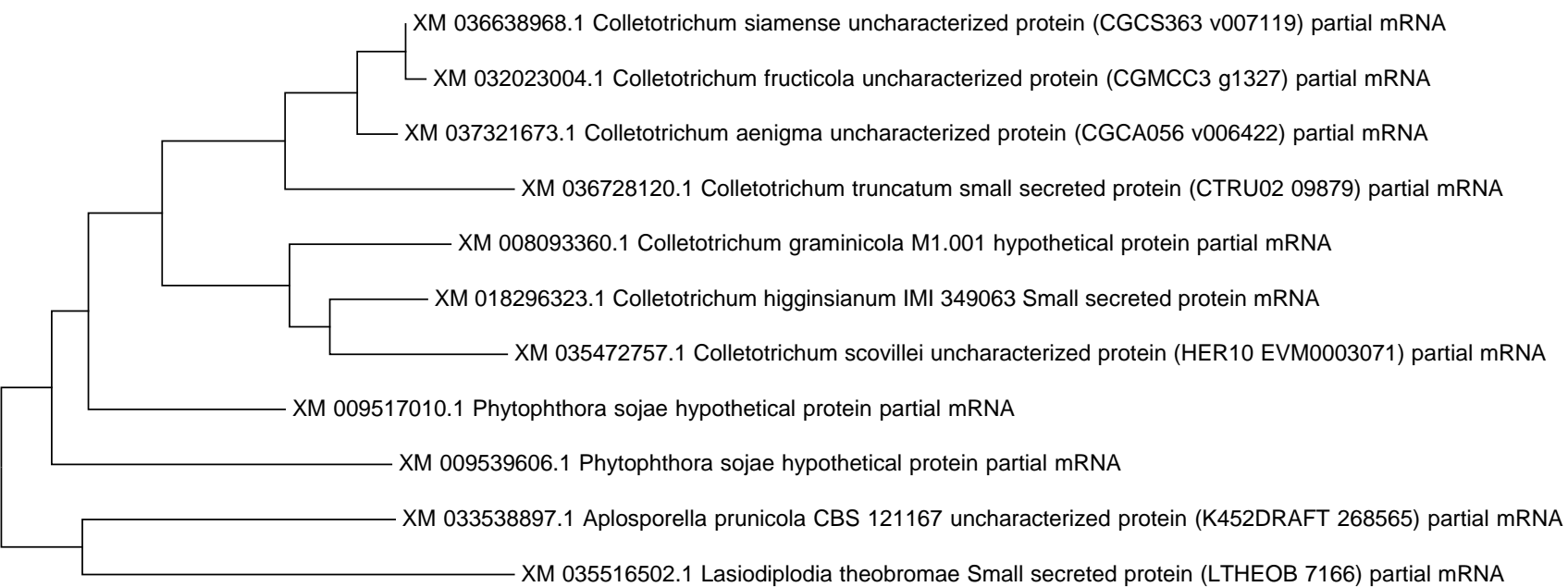

0.050

Supplement: Supplementary Figure 6 — Phylogenetic tree of top 10 sequences with close relationships to CgNPG1. [file Data_Sheet_6.PDF]
